# Postoperative Intra-Abdominal *Clostridium tertium* Infection Following Obstructed Obturator Hernia Repair: A Case Report and Literature Review

**DOI:** 10.3390/pathogens15040348

**Published:** 2026-03-25

**Authors:** Jin Lu, Guanjun Zhan, Zhongjing Meng, Yuchen Zhang, Xiangkai Zhuge

**Affiliations:** 1 Department of Pharmacy, Jiangbei District of Zhongda Hospital Affiliated to Southeast University, Nanjing 210048, China; 13914789051@163.com (J.L.); mengzhongjing@126.com (Z.M.); 18851610175@163.com (Y.Z.); 2 Department of Pharmacy, Nanjing Dachang Hospital, Nanjing 210048, China; 3 Department of Pharmacy, Zhongda Hospital Affiliated to Southeast University, Nanjing 210009, China; 13776653295@163.com; 4 Department of Nutrition and Food Hygiene, School of Public Health, Nantong University, Nantong 226019, China

**Keywords:** *Clostridium tertium*, intra-abdominal infection, obturator hernia, postoperative complication, non-neutropenic host

## Abstract

*Clostridium tertium* is an emerging opportunistic pathogen typically associated with immunocompromised hosts, yet it can also cause serious infections in non-neutropenic individuals. We present a case of postoperative peritonitis and bacteremia caused by *C. tertium* in a non-neutropenic 75-year-old woman following emergency obturator hernia repair. Diagnosis was confirmed by matrix-assisted laser desorption/ionization time-of-flight mass spectrometry (MALDI-TOF MS), and successful treatment was achieved with piperacillin–tazobactam combined with levornidazole alongside surgical source control. A review of 128 cumulative cases (including ours) revealed two distinct patterns: bacteremia in severely neutropenic patients versus a broader spectrum of localized and bloodstream infections in non-neutropenic hosts, often linked to intestinal barrier disruption. Mortality was largely driven by underlying comorbidities and polymicrobial sepsis. These findings indicate that *C. tertium* infection should be considered in non-neutropenic patients with postoperative or gastrointestinal barrier-disruptive infections, especially when there is a poor response to initial empiric therapy. Consequently, in such clinical scenarios, empirical therapy should be guided by its unique resistance pattern, favoring carbapenems, vancomycin, or piperacillin–tazobactam, often combined with a nitroimidazole, alongside urgent source control.

## 1. Introduction

*Clostridium tertium* is an aerotolerant, spore-forming, Gram-positive bacillus, an uncommon but increasingly recognized opportunistic human pathogen. As part of the normal microbiota, it colonizes the oral cavity and gastrointestinal tract (GIT) and is also present in soil. It primarily causes infection in immunocompromised hosts, patients with underlying gastrointestinal disorders, or following abdominal procedures [1]. Unlike many toxigenic *Clostridium* species, *C. tertium* has historically been regarded as non-toxin-producing, although its pathogenic mechanisms are under reevaluation [2].

Its clinical management is complicated by two key characteristics. First, accurate identification is challenging due to its aerotolerant nature, which permits growth under ambient atmospheric conditions and frequently leads to misidentification as *Bacillus*, *Lactobacillus*, or even Gram-negative rods in routine cultures [3,4]. Second, it exhibits an atypical and challenging antimicrobial susceptibility profile. This includes variable susceptibility to metronidazole, frequent resistance to clindamycin and expanded-spectrum cephalosporins [4,5], and, notably, a predictable resistance to many third- and fourth-generation cephalosporins such as ceftriaxone and cefotaxime [6,7]. This specific resistance profile may facilitate its selection and emergence as a breakthrough pathogen in patients receiving empirical therapy with these agents.

*C. tertium* infections have been documented in diverse clinical contexts, including necrotizing soft tissue infections, osteomyelitis, empyema, and ocular infections, typically following compromise of local barriers or direct inoculation. However, due to its diagnostic pitfalls and atypical susceptibility pattern, the full clinical spectrum and optimal management strategies remain incompletely defined. To address this knowledge gap, we report a novel case of postoperative peritonitis and bacteremia due to *C. tertium* in an elderly patient following emergency surgery for an obstructed obturator hernia, and complement it with a comprehensive review of published international case reports and series, including literature in English, Chinese, and other languages. This work aims to delineate the clinical spectrum of *C. tertium* infections, compare its epidemiological patterns across different host populations, and synthesize a contemporary evidence base to guide risk assessment and therapeutic decision-making.

## 2. Case Presentation

A 75-year-old woman presented with acute abdominal pain, distension, nausea, and vomiting one day after a mechanical fall, later developing coffee-ground vomitus. A computed tomography (CT) scan demonstrated a right obturator hernia with small bowel involvement, proximal bowel dilation, intra-abdominal fluid, and pneumatosis, confirming acute small bowel obstruction. Her vital signs were stable on admission, but laboratory tests revealed leukocytosis (white blood cell count 11.75 × 10^9^/L, reference range: 3.5–9.5 × 10^9^/L) with a neutrophilic predominance (89.4%, reference range: 40–75%) and C-reactive protein (42.38 mg/L, reference range: 0–3.0 mg/L). Following preoperative antibiotic prophylaxis, she underwent emergency laparoscopic surgery which included hernia reduction, resection of non-viable bowel, peritoneal lavage, and placement of a drain. Intraoperative hypotension occurred and was managed with vasopressor support, followed by transfer to the intensive care unit (ICU).

Empiric intravenous cefoperazone–sulbactam was initiated for intra-abdominal infection. However, her condition deteriorated on postoperative day 2, necessitating mechanical ventilation. Significant leukocytosis (white blood cell count 18.40 × 10^9^/L, reference range: 3.5–9.5 × 10^9^/L) and rising inflammatory markers (C-reactive protein 84.26 mg/L, reference range: 0–3.0 mg/L, procalcitonin 6.03 ng/mL, reference range: 0–0.5 ng/mL) were observed alongside persistent abdominal tenderness and drainage of purulent fluid. After inoculation of blood and peritoneal drainage samples onto blood agar plates and anaerobic incubation at 37 °C for 48 h, the isolated organism was identified as *C. tertium* using matrix-assisted laser desorption/ionization time-of-flight mass spectrometry (MALDI-TOF MS; Autobio MS-600 instrument (Autobio Diagnostics Co., Ltd., Zhengzhou, China)). Formal antimicrobial susceptibility testing was not performed as standardized anaerobic susceptibility testing protocols were unavailable at our institution. Given the clinical progression despite the initial regimen (cefoperazone–sulbactam) and based on the literature reports indicating the potential efficacy of piperacillin–tazobactam combined with a nitroimidazole against *C. tertium* infections [8,9], the empirical therapy was switched to piperacillin–tazobactam plus levornidazole (a nitroimidazole antimicrobial).

Source control was maintained via the indwelling drain, along with general supportive care in the ICU. As inflammatory markers declined, enteral nutrition was initiated. The patient’s condition gradually improved, followed by normalization of leukocyte count and inflammatory markers. Antibiotics were discontinued after a 10-day course, as the patient achieved clinical resolution (normalized leukocyte count, resolution of abdominal tenderness, and negative repeat cultures) and in accordance with recommendations for intra-abdominal infections caused by anaerobic pathogens. She was subsequently transferred to a general ward and made a full recovery, and was discharged home two weeks later.

### Literature Review Methodology

To complement our case findings and summarize the existing evidence on *C. tertium* infections, we conducted a comprehensive, non-systematic narrative review of the literature. We performed literature searches in PubMed/MEDLINE, Embase, Web of Science, Google Scholar, CNKI, and Wanfang Database for articles published up to December 2025, using search terms including “*Clostridium tertium*,” “*C. tertium* infection,” and “*C. tertium* bacteremia.” Additional references were identified through bibliography screening. We included published case reports and series describing human infections with microbiologically confirmed *C. tertium.* Reviews, non-human studies, and duplicate reports were excluded. Data on patient demographics, clinical features, management, and outcomes were extracted by one author and cross-checked by another. Therefore, given the narrative and integrative aim of this review, a formal systematic review protocol was not preregistered, and a risk of bias assessment of individual case reports was not performed.

## 3. Discussion

### 3.1. Clinical and Epidemiological Spectrum

This report describes a novel presentation of postoperative peritonitis and bacteremia caused by *C. tertium* following emergency surgery for an obstructed obturator hernia. This case, involving an elderly patient with acute bowel compromise but without classic neutropenia, expands the recognized clinical spectrum beyond severely immunocompromised hosts and underscores its role as an opportunistic pathogen in settings of gastrointestinal barrier disruption. To contextualize our findings, we conducted a comprehensive narrative literature review as detailed in Section Literature Review Methodology, which included for analysis five case series (74 patients) and 44 case reports (53 patients), in addition to our index case.

Our analysis reveals two distinct epidemiological patterns (summarized in Table 1 and Table 2). Case series data depict a highly uniform profile: infections occur almost exclusively in severely neutropenic patients with hematologic malignancies (98.6%, 73/74), universally present as bacteremia (100%), and are strongly linked to prior broad-spectrum antibiotic exposure [10]. This clustering [11] points to an endogenous, gut-derived pathogenesis facilitated by chemotherapy-induced mucosal injury, impaired immunity, and antibiotic selective pressure (e.g., from third-generation cephalosporins [12]).

In contrast, aggregated individual case reports present a more diverse picture. While bacteremia remains common (61.1%, 33/54), localized infections (intra-abdominal, soft tissue/bone, central nervous system) are frequently reported. Underlying conditions are broader, including cirrhosis, trauma, and postoperative states, with a lower prevalence of neutropenia (40.7%, 22/54), indicating increasing recognition in non-neutropenic hosts with local barrier breakdown [1,31]. Consequently, the isolation of *C. tertium* from sterile sites should prompt evaluation for occult gastrointestinal pathology or anatomical communications with the gut [23].

The pathogenesis of *C. tertium* infection is tightly linked to barrier disruption and bacterial translocation. Key risk factors include intestinal mucosal injury, neutropenia, and prior exposure to β-lactam antibiotics, particularly third-generation cephalosporins [11,13]. In neutropenic patients, chemotherapy-induced damage facilitates systemic spread, leading to breakthrough bacteremia [10,19]. In non-neutropenic hosts, conditions like cirrhosis, inflammatory bowel disease, or recent surgery serve as the portal of entry [13], as seen in spontaneous bacterial peritonitis [2,8], post-surgical infections [38], and cases following acute colitis or pancreatitis [3,4,35]. Recent cases of ocular infection [47] and empyema [48] further exemplify mechanisms of direct inoculation or contiguous spread.

Despite its historical classification as non-toxigenic, emerging genomic insights reveal putative virulence factors (e.g., EndoA interferase, hemolytic phospholipase C) that may contribute to tissue damage and severe localized infections [21,23]. Clinical severity is reflected in significant rates of septic shock and mortality (ranging from ~14% to 71% in compiled series), often driven by underlying host conditions or polymicrobial sepsis [10,13]. These findings underscore that *C. tertium*, while often of low individual virulence, can trigger life-threatening illness in compromised hosts.

### 3.2. Diagnostic Challenges and Advances

Accurate identification of *C. tertium* has been historically challenging due to its aerotolerance, variable Gram-staining (often leading to misidentification as *Bacillus* or *Lactobacillus*), and ability to form spores [20,21,22]. The advent of MALDI-TOF mass spectrometry has revolutionized its identification, as exemplified in our case, providing rapid and accurate results that overcome traditional phenotypic pitfalls [7]. In resource-limited settings lacking MALDI-TOF MS, molecular techniques such as 16S rRNA gene sequencing provide a valuable diagnostic alternative, enabling precise species-level identification.

### 3.3. Management Principles and Antimicrobial Therapy

The management of *C. tertium* infection is fundamentally predicated on the concurrent application of two principles: prompt and definitive source control, and antimicrobial therapy deliberately selected to overcome its characteristic resistance profile [27,38,40]. For invasive infections, this translates to immediate surgical interventions such as debridement for necrotizing soft tissue infections, drainage for empyema or abscesses, and removal of infected devices.

The selection of antimicrobial agents must be guided by a clear understanding of the pathogen’s susceptibility patterns, as synthesized from 38 clinical isolates in Table 3. It is important to note that the data in Table 3 span a long publication period (1963–2025), and temporal evolution in susceptibility testing methods and breakpoints may influence the interpretation of these aggregated rates. Carbapenems (imipenem, meropenem, ertapenem) and vancomycin demonstrated 100% susceptibility, solidifying their role as cornerstone therapeutic options for serious infections [12,35]. In stark contrast, therapeutic regimens should deliberately avoid clindamycin and expanded-spectrum cephalosporins such as ceftriaxone and cefepime, given their associated high resistance rates of 90.0% and 85.7%, respectively [7,12]. Despite the reported susceptibility of piperacillin–tazobactam being 76.5% (Table 3), its combination with a nitroimidazole (e.g., levornidazole or metronidazole) has been reported as a clinically used strategy, as demonstrated in our case and supported by several reports [8,9]. This approach may be particularly rational in intra-abdominal infections to ensure broad anaerobic coverage and address frequent polymicrobial involvement, although robust in vitro synergy data specific to *C. tertium* are lacking.

A significant area of therapeutic uncertainty lies in the variable activity of other agents. Susceptibility to penicillins and β-lactam/β-lactamase inhibitor combinations is inconsistent. Perhaps most critically for empirical anaerobic coverage, metronidazole demonstrates a non-negligible rate of non-susceptibility (11.1%), challenging its reliability when *C. tertium* is suspected [4,27].

In the present case, the initial clinical progression despite therapy with cefoperazone–sulbactam may be explained by the organism’s inherent resistance profile [11,22]. The escalation to piperacillin–tazobactam combined with levornidazole represented a pragmatic strategy, utilizing an alternative β-lactam/β-lactamase inhibitor while providing reinforced anaerobic coverage based on the literature reports of efficacy [8,9]. For severe infections, an initial combination therapy is often employed, typically followed by a total treatment course of 14 to 28 days. Ultimately, successful outcomes depend on integrating rapid diagnosis, antimicrobial therapy that strategically bypasses predictable resistances, and uncompromising source control.

## 4. Conclusions

We report a novel case of *C. tertium* postoperative peritonitis and bacteremia following emergency obstructed hernia repair in a non-neutropenic patient. The successful outcome, achieved through timely surgical source control and literature-guided antimicrobial therapy, underscores the manageability of this infection when its unique resistance profile is considered. Our integrated analysis of 128 cases delineates two principal epidemiological patterns: breakthrough bacteremia in neutropenic hosts and a broader spectrum of localized infections in non-neutropenic hosts, both frequently associated with gastrointestinal barrier compromise.

Key clinical implications derived from this review include: (1) empirical antimicrobial therapy should avoid expanded-spectrum cephalosporins and clindamycin, favoring agents like carbapenems, vancomycin, or piperacillin–tazobactam, with caution regarding metronidazole non-susceptibility; (2) prompt and adequate source control is critical; and (3) recognizing that patient outcomes are significantly influenced by underlying comorbidities and polymicrobial sepsis, necessitating holistic management.

This study has several limitations. Foremost, the absence of formal antimicrobial susceptibility testing for the index isolate precludes definitive confirmation of its resistance pattern and limits its contribution to local epidemiology. Furthermore, because the literature synthesis spans over six decades, it inherently incorporates heterogeneity in data quality, reporting standards, and susceptibility testing methodologies. The analysis is also subject to the inherent biases of published case reports and series, such as publication bias and selective reporting. Taken together, these constraints confined our study to a comprehensive narrative synthesis, precluding a formal meta-analysis.

## Figures and Tables

**Table 1:** Clinical characteristics and outcomes of *Clostridium tertium* infections in immunocompromised hosts: summary of case series.

Authors/ Publication Year	No. of Cases	Age Range (Years)	Sex (M:F)	Clinical Features	Predisposing Factors	Effective Antimicrobial Therapy	Outcome
Thaler M et al., 1986 [6]	10	9–64	6:4	Bacteremia (7), Intra- abdominal infection /Perirectal cellulitis (5), Intra-abdominal abscess (2), Pleural empyema (1), Fever	Hematologic malignancies (9/10), Neutropenia, Prior broad- spectrum antibiotics	Vancomycin (added in 5 cases), Chloramphenicol (added in 4 cases)	6 survived; 4 died (underlying disease progression, polymicrobial sepsis)
Speirs G et al., 1988 [12]	18	14–79	10:8	bacteremia (18), Abdominal pain/Diarrhea/Rectal bleeding (15), Perianal cellulitis (3)	Hematologic malignancies (18), Neutropenia (18), Prior 3rd-gen cephalosporin use	Vancomycin (used in 14/18 patients)	14 survived; 4 died (polymicrobial bacteremia, septic shock)
Valtonen M et al., 1990 [11]	7	19–58	3:4	Bacteremia (7), Perirectal/ Cecal cellulitis (3), Febrile neutropenia	Acute leukemia (7), Neutropenia (7), Recent 3rd-gen cephalosporin therapy	Vancomycin, (added to regimen in 5 patients)	6 survived; 1 died (disseminated aspergillosis, pulmonary embolism)
Miller DL et al., 2001 [13]	32	16–75	20:12	Bacteremia (32), Febrile episode (32), Gastrointestinal symptoms (19)	Neutropenia (29/32), Hematologic malignancies, Chemotherapy, β-lactam exposure	Not specified per case	28 survived; 4 died within 1 week (underlying leukemia, polymicrobial infection, bowel ischemia)
Shah S et al., 2016 [10]	7	36–82	4:3	Bacteremia (7), Febrile neutropenia (7), Gastrointestinal complaints (4)	AML/MDS (7), Neutropenia (7), Chemotherapy	Vancomycin (used in 6/7 patients), Ciprofloxacin, Metronidazole (used in combination)	2 survived; 5 died (progression of hematologic malignancy)

Note: M: male, F: female, AML: acute myeloid leukemia, MDS: myelodysplastic syndrome, ALL: acute lymphoblastic leukemia, CML: chronic myeloid leukemia, NEC: necrotizing enterocolitis, SBP: spontaneous bacterial peritonitis, ESRD: end-stage renal disease.

**Table 2:** Clinical data summary of 53 patients with *Clostridium tertium* infection.

Authors/ Publication Year	Age/ Gender	Clinical Symptoms	Specimen	Possible Risk Factors	Probable Source	Antimicrobial Therapy Following *Clostridium tertium* Diagnosis	Course of Treatment
King BM et al., 1963 [14]	7/M	Fever, cough, leukocytosis, lung consolidation	Blood	Influenza-like illness	Respiratory	Penicillin	Survived
	7/M	Fever, leukocytosis, lung consolidation	Blood	Influenza-like illness	Respiratory	Penicillin, tetracycline	Survived
Butler T et al., 1982 [15]	42/F	Jaundice, ascites, fever	Ascitic fluid	Cirrhosis, prior antibiotics	GIT	Cefoxitin	Survived
Weiser J et al., 1987 [16]	60/F	Fever, bowel obstruction	Blood	Severe retardation, surgery	NS	Penicillin	Survived
Johnson JR et al., 1988 [17]	48/M	Fever, respiratory distress, leukocytosis	Blood, sputum	Alcohol withdrawal, aspiration pneumonia	Aspiration pneumonia	Cefazolin	Survived
Leichtman DA et al., 1989 [18]	72/F	Fever, confusion, hearing deficit	Blood	CML, severe leukopenia	Middle ear infection	Cefazolin	Survived
Brown EA et al., 1989 [19]	29/M	Fever, neutropenia	Blood	ALL, chemotherapy	GIT	Vancomycin, clindamycin	Survived
Lew JF et al., 1990 [20]	8/F	Periorbital swelling, frontal abscess	Swab, brain tissue	Penetrating injury, soil contamination	Soil/wound	Penicillin G	Survived
Coleman N et al., 1993 [21]	15/M	Diarrhea, abdominal pain, neutropenia	Blood, feces	ALL relapse, neutropenia	GIT	Ciprofloxacin, vancomycin	Survived
Gosbell IB et al., 1996 [22]	19/F	Fever, abdominal pain, neutropenia	Blood	ALL relapse, neutropenia	GIT	Metronidazole, vancomycin	Survived
	57/F	Diarrhea, fever, abdominal sepsis	Blood	Ulcerative colitis, intra-abdominal sepsis	Intra-abdominal sepsis	Ciprofloxacin and metronidazole after ticarcillin/ clavulanate and gentamicin	Survived
Kourtis AP et al., 1997 [23]	12/F	Headache, fever, meningitis	CSF	Congenital fistulae, prior antibiotics	GIT/ genital tract	Vancomycin, metronidazole	Survived
Steyaert S et al., 1999 [24]	65/M	Fever, diarrhea, neutropenia	Blood	AML, neutropenia	GIT	Vancomycin	Survived
	55/M	Fever, diarrhea, neutropenia	Blood	ALL, neutropenia	GIT	Vancomycin	Survived
Gredlein CM et al., 2000 [25]	37/M	Foot edema, warmth, erythema	Synovial fluid	Penetrating trauma	Soil/trauma	Clindamycin, penicillin G	Survived
Miller DL et al., 2001 [13]	28/M	Abdominal pain, diarrhea	Blood	Crohn’s disease	GIT	Ciprofloxacin, clindamycin	Survived
Cheah FC et al., 2001 [26]	Preterm/M	Feeding intolerance, abdominal distension	CSF	Prematurity, NEC	GIT	Penicillin, metronidazole	Died (catheter-related septicemia)
Ray P et al., 2003 [27]	58/M	Ulcers, fever, leg gangrene	Necrotic tissue	Alcoholism, lymphoma, chemotherapy	Soil/GIT	Metronidazole, vancomycin, imipenem	Survived
	40/M	Leg gangrene, fever	Necrotic tissue	Trauma	Soil/wound	Penicillin, metronidazole	Survived
Séverin A et al., 2005 [3]	61/F	Abdominal pain, septic shock	Abdominal fluid, blood	Pancreatitis, prior antibiotics	Soil/GIT	Ciprofloxacin, piperacillin/ tazobactam	Died (nosocomial pneumonia)
Tappe D et al., 2005 [28]	51/F	Abdominal pain, septic shock	Blood	Ileus, postoperative state	GIT	Mezlocillin, metronidazole	Died (septic shock with multi-organ failure)
Leegaard TM et al., 2005 [4]	53/M	Fever, neutropenia	Blood	AML, neutropenia	GIT	Imipenem	Survived
	33/F	Fever, pancytopenia	Blood	MDS, stem cell transplant	GIT	Meropenem	Died (multi-organ failure)
	12/F	Fever, neutropenia	Blood	ALL relapse, neutropenia	GIT	Gentamicin, amoxicillin	Survived
Del Rosario- Quintana C et al., 2006 [29]	80/M	Jaundice, ascites	Ascitic fluid	Cirrhosis, nursing home	GIT	Imipenem	Survived
Fujitani S et al., 2007 [30]	51/M	Skin abscesses, myonecrosis	Wound material	IV drug use, skin popping	Heroin/skin	Amoxicillin/ clavulanate and metronidazole after ampicillin/ sulbactam and metronidazole	Survived
Vanderhofstadt M et al., 2010 [31]	51/M	Asymptomatic bacteremia	Blood	AML, pancytopenia	GIT	Amoxicillin/ clavulanate, ceftazidime	Survived
	23/F	Fever, leukopenia	Blood	Lymphoma, chemotherapy	GIT	Amoxicillin/clavulanate, amikacin, ceftazidime	Survived
Steensma EA et al., 2011 [32]	39/M	Cellulitis, myonecrosis	Tissue	Diabetes, IV drug use	Skin/environment	Penicillin G	Survived
Salvador F et al., 2013 [7]	47/F	Fever, abdominal pain, neutropenia	Blood	ALL, chemotherapy	GIT	Meropenem, vancomycin	Survived
Virot E et al., 2015 [33]	40/M	Knee pain, bone infection	Bone tissue	Shrapnel injury	Soil/wound	Amoxicillin, pristinamycin	Survived
You M-J et al., 2015 [5]	44/F	Fever, leukopenia	Blood	Herbicide ingestion	GIT	Ertapenem, metronidazole	Survived
Joichi Y et al., 2016 [34]	50s/M	Shock, hypothermia	Blood	NS	GIT	Piperacillin/ tazobactam	Died (septic shock)
	60s/M	Fever, neutropenia	Blood	AML, chemotherapy	GIT	Meropenem, vancomycin	Survived
Chalhoub V et al., 2016 [35]	54/F	Abdominal pain, septic shock	Blood	Acute colitis	GIT	Ampicillin, imipenem, vancomycin	Survived
Nambiar PH et al., 2016 [1]	53/F	Abdominal pain, abscess	Blood, abscess fluid	Pancreatic cancer, surgery	GIT	Meropenem	Survived
González-Alad MJ et al., 2016 [36]	6/NS	Fever, neutropenia	Blood	AML, stem cell transplant	GIT	Vancomycin	Survived
Zhang AP et al., 2016 [37]	52/M	Headache, meningitis	CSF	Scalp trauma	External environment	Vancomycin	Survived
Alroumi F et al., 2016 [38]	75/M	Dyspnea, empyema	Pleural fluid	Abdominal surgery, steroids	GIT	Clindamycin, vancomycin	Survived
Sutton SS et al., 2017 [2]	60/M	Abdominal pain, ascites	Ascitic fluid	Cirrhosis, hernia repair	GIT	Vancomycin, ciprofloxacin, metronidazole	Survived
Cong PS et al., 2018 [39]	69/M	Abdominal pain, obstruction	Blood	Intestinal volvulus, surgery	GIT	Imipenem, metronidazole	Survived
Barakat M et al., 2018 [40]	70/M	Liver abscess	Abscess fluid	Diverticulosis, COPD	GIT	Piperacillin/ tazobactam, metronidazole	Survived
Wazir M et al., 2019 [8]	62/M	Ascites, encephalopathy	Blood	Cirrhosis, SBP	GIT	Meropenem, vancomycin	Died (shock and respiratory failure)
Milano V et al., 2019 [41]	43/M	Hepatic abscess	Blood	Appendectomy, peritonitis	GIT	Ertapenem	Survived
Li J et al., 2019 [42]	55/F	Osteomyelitis	Bone tissue, secretions	Open fracture	Trauma/wound	Metronidazole	Survived
	44/F	Osteomyelitis	Bone tissue, secretions	Open trauma	Trauma/wound	Metronidazole	Survived
Morikawa K et al., 2021 [43]	1d/F	Sepsis, abdominal distension	Blood, ascitic fluid	Ileal atresia, perforation	GIT	Meropenem, vancomycin	Survived
Bonda S et al., 2022 [44]	79/M	Septic shock, peritonitis	Blood	Colon perforation, alcoholism	GIT	Meropenem	Survived
Saad E et al., 2022 [45]	66/F	Septic shock, diverticulitis	Blood	COVID-19, diabetes, steroids	GIT	Meropenem, metronidazole, amoxicillin/ clavulanate	Survived
Lamberto Y et al., 2023 [9]	48/M	Peritonitis, septic shock	Blood, abdominal fluid	Cirrhosis, hernia surgery	GIT	Piperacillin/ tazobactam	Died (septic shock)
Duan Y et al., 2023 [46]	68/F	Diarrhea, fever	Blood	Pancreatic cancer, chemotherapy	GIT	Piperacillin/ tazobactam	Survived
Zhu W et al., 2024 [47]	54/M	Ocular trauma, infection	Eye secretions	Explosion injury	External environment	Vancomycin, levofloxacin	Survived
Lovering E et al., 2025 [48]	60s/M	Empyema, pleural effusion	Pleural fluid	ESRD, prior peritonitis	GIT	Metronidazole	Died after 4 months (respiratory distress)

Note: Abbreviations as in Table 1; GIT: gastrointestinal tract, CSF: cerebrospinal fluid, COPD: chronic obstructive pulmonary disease, NS: not specified.

**Table 3:** Summary of antimicrobial susceptibility patterns in reported cases of *Clostridium tertium* infection (1963–2025).

Antimicrobial Class & Agent	Sensitive (S)	Intermediate + Resistant	Total Isolates Tested	Susceptibility Rate (S%)
Penicillins (Penicillin G, Ampicillin)	16	8	24	66.7
β-Lactam/β-Lactamase Inhibitors (e.g., Amoxicillin–Clavulanate, Piperacillin–Tazobactam)	13	4	17	76.5
Cephalosporins				
1st/2nd Generation (e.g., Cefazolin, Cefalotin)	4	3	7	57.1
Cephamycins (e.g., Cefoxitin, Cefotetan)	10	1	11	90.9
3rd/4th Generation (e.g., Ceftriaxone, Cefepime)	2	12	14	14.3
Carbapenems (Imipenem, Meropenem, Ertapenem)	19	0	19	100.0
Vancomycin	20	0	20	100.0
Metronidazole	24	3	27	88.9
Clindamycin	2	18	20	10.0
Fluoroquinolones (e.g., Cliprofloxacin, Levofloxacin)	8	1	9	88.9
Aminoglycosides (e.g., Gentamicin, Amikacin)	0	5	5	0.0

Note: Data are summarized from 38 isolates with susceptibility reported for agents tested in ≥5 cases.

## Data Availability

All data generated or analyzed during this study are included in this published article. Further inquiries can be directed to the corresponding author.
